# Supplementary material for: F18:A-:B1 Plasmids Carrying blaCTX-M-55 Are Prevalent among Escherichia coli Isolated from Duck–Fish Polyculture Farms
Source: Antibiotics (Basel). 2023 May 25;12(6):961. doi: 10.3390/antibiotics12060961 (PMC10295773; doi:10.3390/antibiotics12060961)
Supplement: Supplementary file 1 [file antibiotics-12-00961-s001.zip › antibiotics-2409227-SI.pdf]

Supplementary materials

Table S1: Sample and isolate information from different sources

Sample Source	Farm A				Farm B				Farm C				Farm D			
	Water	Soil	Foliage	Duck fecal	Water	Soil	Foliage	Duck fecal	Water	Soil	Foliage	Duck fecal	Water	Soil	Foliage	Duck fecal
Sample Number (n)	102	81	59	199	106	129	0	158	138	152	20	300	150	114	0	300
CTX-M-1G-positive Strains (n)	2	2	1	19	7	1	0	21	7	0	0	54	18	4	0	60
bla$_{CTX-M-55}$ Positive Strains (n)	2	2	1	17	7	1	0	14	7	0	0	51	15	4	0	56
bla$_{CTX-M-55}$ Positive Rate (%)	4.25	8.0	25.0	13.0	13.2	4.0	0	15.4	10.1	0	0	28.8	21.1	16.7	0	38.4

Table S2: The MIC results of 177 *bla*$_{CTX-M-55}$-positive *E. coli* strains

Antibiotics	Resistance breakpoint	MIC_{50} （μg/mL）	MIC_{90} （μg/mL）	MIC range （μg/mL）	Resistance rate (%)
Cefotaxime	≥4	128	256	4- > 512	100
Ceftiofur	≥8	128	512	0.25- > 512	98.3
Meropenem	≥4	0.015	0.03	0.015-8	3.38
Ceftazidime	≥16	8	32	0.125- > 512	31.07
Amikacin	≥64	2	4	< 0.5- > 512	5.64
Ciprofloxacin	≥1	16	128	0.25- > 512	96.04
Tigecycline	≥2	0.5	1	0.06-8	5.64
Florfenicol	≥16	256	512	4- > 512	95.4
Colistin	≥4	0.5	8	0.25-16	35.0
Fosfomycin	≥256	2	512	1-1024	29.9

Table S3: Bacterial information and antimicrobial resistance profiles

Strain name	Sources	IncF plasmid type	Transfer frequency	*bla*$_{CTX-M-55}$ location	*bla*$_{CTX-M-55}$ genetic environment	Resistance phenotype
ND104	Fecal swab	F33:A-:B-	-	Plasmid	Type III	CTX/CTF/CIP/FLF/CL/FOS
RC2	Water	F33:A-:B-	-	Plasmid	Type III	CTX/CTF/CIP/FLF/CL/FOS
DT6	Soil	F33:A-:B-	2.41×10^{-4}	Plasmid	Type II	CTX/CTF/CIP/FLF/FOS
KS25	Fecal swab	F33:A-:B-	1.67×10^{-4}	Plasmid	Type III	CTX/CTF/CIP/FLF/CL
RDW3	Water	F33:A-:B-	1.18×10^{-1}	Plasmid	Type III	CTX/CTF/CIP/FLF/FOS
DW6	Water	F33:A-:B-	2.6×10^{-5}	Plasmid	Type II	CTX/CTF/CTZ/CIP/FLF/CL
JDS19	Fecal swab	F33:A-:B-	1.12×10^{-2}	Plasmid	Type II	CTX/CTF/CIP/
RDW5	Water	F33:A-:B-	5.94×10^{-2}	Plasmid	Type III	CTX/CTF/CIP/FLF/FOS
B1S17	Water	F33:A-:B-	1.63×10^{-1}	Plasmid	Type III	CTX/CTF/CIP/FLF/FOS
UC8	Fecal swab	F33:A-:B-	3.48×10^{-2}	Plasmid	Type II	CTX/CTF/CTZ/CIP/FLF/FOS
JDS24	Fecal swab	F18:A-:B1	6.39×10^{-2}	Plasmid	Type II	CTX/CTF/CIP
B1S11	Water	F18:A-:B1	-	Plasmid	Type IV	CTX/CTF/CIP/FLF/FOS
TC5	Fecal swab	F18:A-:B1	-	Plasmid	Type I	CTX/CTF/FLF/CL
UD5	Fecal swab	F18:A-:B1	-	Plasmid	Type I	CTX/CTF/CIP/FLF/CL
RD1	Fecal swab	F18:A-:B1	-	Plasmid	Type I	CTX/CTF/CIP/FLF/CL
B1W1	Water	F18:A-:B1	-	Plasmid	Type I	CTX/CTF/CIP/FLF/CL
UD9	Fecal swab	F18:A-:B1	-	Plasmid	Type I	CTX/CTF/CIP/FLF/CL
D7	Fecal swab	F18:A-:B1	-	Plasmid	Type I	CTX/CTF/CIP/FLF/CL
PBS4	Fecal swab	F18:A-:B1	1.54×10^{-2}	Plasmid	Type II	CTX/CTF/CIP/FLF
PBS5	Fecal swab	F18:A-:B1	1.91×10^{-2}	Plasmid	Type II	CTX/CTF/CIP/FLF
PBS10	Fecal swab	F18:A-:B1	4.06×10^{-2}	Plasmid	Type II	CTX/CTF/CIP/FLF
PBS13	Fecal swab	F18:A-:B1	1.99×10^{-2}	Plasmid	Type II	CTX/CTF/CIP/FLF
C6	Fecal swab	F18:A-:B1	-	Plasmid	Type II	CTX/CTF/CIP/FLF
PBS21	Fecal swab	F18:A-:B1	1.53×10^{-2}	Plasmid	Type II	CTX/CTF/CIP/FLF

D52	Fecal swab	F18:A-:B1	-	Chromosome	Type I	CTX/CTF/CIP/FLF
C28	Fecal swab	F18:A-:B1	-	Plasmid	Type I	CTX/CTF/CTZ/CIP/FLF/CL/FOS
MA121	Fecal swab	F18:A-:B1	2.47×10^{-2}	Plasmid	Type III	CTX/CTF/CIP/FLF
D50	Fecal swab	F18:A-:B1	-	Chromosome	Type I	CTX/CTF/CTZ/CIP/FLF
NDT16	Soil	F18:A-:B1	-	Chromosome	Type I	CTX/CTF/CIP/FLF
D51	Fecal swab	F18:A-:B1	-	Chromosome	Type I	CTX/CTF/CIP/FLF
D38	Fecal swab	F18:A-:B1	-	Chromosome	Type I	CTX/CTF/CIP/FLF
D49	Fecal swab	F18:A-:B1	-	Chromosome	Type I	CTX/CTF/CIP/FLF
D33	Fecal swab	F18:A-:B1	-	Chromosome	Type I	CTX/CTF/CIP/FLF
KS22	Fecal swab	F18:A-:B1	2.47×10^{-2}	Plasmid	Type II	CTX/CTF/CIP/FLF/CL/FOS
KW21	Water	F18:A-:B1	8.60×10^{-3}	Plasmid	Type III	CTX/CTF/CIP/FLF/CL/FOS
D2	Fecal swab	F18:A-:B1	-	Chromosome	Type I	CTX/CTF/CIP/FLF/CL
UD1	Fecal swab	F18:A-:B1	8.35×10^{-5}	Plasmid	Type III	CTX/CTF/CIP/FLF
C44	Fecal swab	F18:A-:B1	-	Chromosome	Type I	CTX/CTF/MRO/CTZ/AMI/CIP/FLF/CL/FOS
C48	Fecal swab	F18:A-:B1	-	Chromosome	Type I	CTX/CTF/CTZ/CIP/FLF/FOS
MD91	Fecal swab	F18:A-:B1	-	Plasmid	Type I	CTX/CTF/CIP/FLF/CL
A44	Fecal swab	F18:A-:B1	2.6×10^{-5}	Plasmid	Type I	CTX/CTF/CTZ/FLF
PBS14	Fecal swab	F18:A-:B1	-	Plasmid	Type III	CTX/CTF/CIP/FLF
C42	Fecal swab	F18:A-:B1	-	Plasmid	Type IV	CTX/CTF/CTZ/CIP/FLF
D5	Fecal swab	F18:A-:B1	4.65×10^{-1}	Plasmid	Type II	CTX/CTF/CIP/FLF/CL/FOS
D25	Fecal swab	F18:A-:B1	-	Chromosome	Type I	CTX/CTZ/CIP/FLF/CL/FOS
RDW9	Water	F18:A-:B1	-	Plasmid	Type I	CTX/CTF/CIP/FLF
BT2	Soil	F18:A-:B1	4.56×10^{-6}	Plasmid	Type IV	CTX/CTF/CIP/FLF

CTX, cefotaxime; CTF, ceftiofur; CTZ, ceftazidime; MEM, meropenem; FLF, florfenicol; CIP, ciprofloxacin; FOS, fosfomycin; CL, colistin; AMK, amikacin; TIG, tigecycline.

Table S4: Characteristics of sequenced strains and obtained F18:A-:B1 plasmids

Strain name	Source	Collection year	*bla*CTX-M-55 transferability	Plasmid name	Plasmid Size	GC Content
KW21	Water	2018	Yes	pKW21	179,817 bp	52%
B1W1	Water	2019	No	pB1W1	108,673 bp	50%
B1S11	Fecal	2019	No	pB1S11	151,146 bp	50%
PBS4	Fecal	2019	Yes	pPBS4	137,545 bp	51%

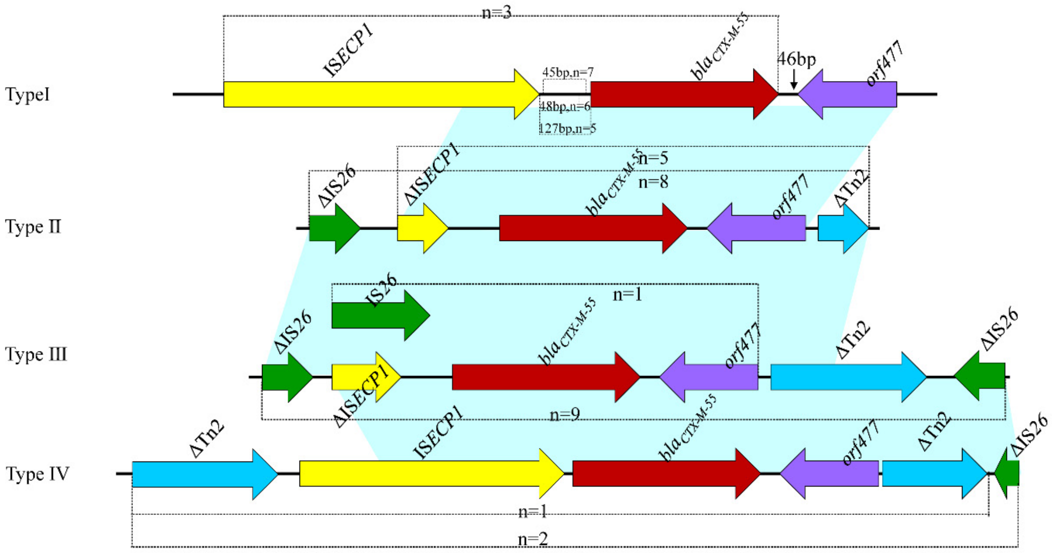

Figure S1: Comparison of four types of *bla*CTX-M-55 genomic contexts in 47 strains

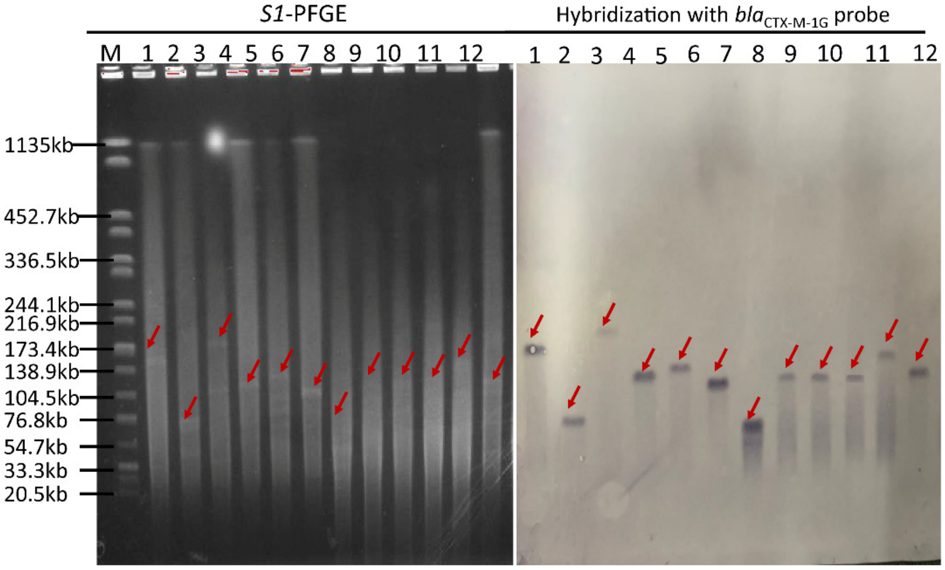

Figure S2 Pulsed field gels of S1 digested genomic DNA and Southern blot in-gel hybridization with $bla_{CTX-M-55}$ probe. M: H9812, 1-12: $bla_{CTX-M-55}$-positive transconjugants MA121, D5, KW21, PBS4, KS22, PBS10, BT2, PBS5, PBS13, PBS21, UD1 and A44.
